# Infants With Low Birth Weight Have Larger Adipocytes During Early Childhood

**DOI:** 10.1111/ijpo.70123

**Published:** 2026-06-12

**Authors:** Tetsuro Murakawa, Yuya Nakano, Akio Ebata, Yoshiyuki Hasebe, Keiko Nagahara, Katsumi Mizuno

**Affiliations:** ^1^ Department of Pediatrics Showa Medical University Tokyo Japan

**Keywords:** adipocyte, early childhood, hypertrophy, low birth weight infants

## Abstract

**Background:**

Infants with low birth weight are at increased risk of developing insulin resistance later in life.

**Objectives:**

To evaluate differences in adipocyte size during early childhood between infants with low birth weight and infants born appropriate for gestational age at full‐term.

**Methods:**

This cross‐sectional study included 92 children (77 infants born appropriate for gestational age at full‐term and 15 with low birth weight), aged 0.8–5.2 years, scheduled for surgery. Adipose tissue samples were obtained intraoperatively, fixed in osmic acid, and assessed for mean adipocyte diameter after adipocyte separation.

**Results:**

Despite a similar body mass index, the low‐birth‐weight group had larger mean adipocyte diameters during early childhood than the term appropriate‐for‐gestational‐age group (87.0 μm vs. 74.7 μm). Multiple regression analysis revealed that low birth weight and higher body mass index at surgery were significant determinants of larger mean adipocyte diameter (*p* < 0.001). Mean adipocyte diameter was not associated with haematological parameters, including insulin, adiponectin, and leptin levels.

**Conclusion:**

During early childhood, infants with low birth weight had larger adipocytes than infants born appropriate for gestational age at term. Further studies are required to determine whether this observation is associated with insulin resistance development later in life.

## 1 Introduction

Accumulating evidence has revealed that infants with low birth weight (LBW) have an increased risk of developing insulin resistance and morbidities, such as diabetes mellitus and cardiovascular disease, later in life [1, 2]. Additionally, early postnatal factors, including feeding method (breastfeeding or infant formula) [3] and complementary food (timing and content) [4], have been identified as key determinants influencing the risk of obesity. The developmental origins of health and disease concept suggests that a mismatch between intrauterine and postnatal life may be associated with these risks. Infants with LBW who experience malnutrition during intrauterine life may acquire a thrifty phenotype characterized by enhanced energy efficiency through altered endocrine and metabolic mechanisms. When exposed to excessive nutrition after birth, these infants are more likely to develop obesity and have an increased risk of glucose intolerance later in life [5, 6].

Although numerous animal studies have supported these mechanisms [7], a notable contradiction arises when applied to humans, as several studies have reported a linear positive relationship between birth weight and obesity risk in adults [8]. Therefore, in individuals born with LBW, mechanisms other than obesity may contribute to their elevated risk of insulin resistance, in contrast to human adults with metabolic syndrome, in whom obesity is typically a key factor. Infants with LBW have been reported to develop increased visceral fat [9, 10] and altered body composition [11, 12], characterized by lower lean body mass and higher body fat percentages later in life. These changes may contribute to the development of insulin resistance in infants with LBW, even in the absence of overt obesity. Recent animal studies have demonstrated that myogenic insulin resistance is associated with reduced lean body mass in a non‐obese hyperglycaemic mouse model [13] and a novel fetal growth restriction model [14].

Fat mass can expand through an increase in the average adipocyte size (hypertrophy) and/or number (hyperplasia; defined as an increase in cell number through proliferation and differentiation of adipocyte precursor cells), both of which are considered important factors in influencing adipose tissue accumulation and insulin sensitivity [15]. The body stores excess energy as triglycerides in adipose tissue, resulting in adipocyte hypertrophy. Larger adipocytes alter the hormonal profile of adipokines, which is an essential factor in the development of insulin resistance in individuals with obesity [16, 17]. Conversely, individuals with fewer adipocytes may be leaner as adults with an increased risk of glucose intolerance, similar to individuals with lipoatrophic diabetes mellitus [18].

During fetal development, the adipose tissue rapidly expands, predominantly due to adipocyte hyperplasia, with an increase in the number of small adipocytes, particularly during the second half of fetal life. In contrast, adipose tissue accumulates during the first 12 months of life, primarily due to adipocyte hypertrophy [19]. The total number of adipocytes increases during childhood and adolescence and plateaus in adulthood, remaining unchanged even after considerable weight loss following bariatric surgery [20]. Furthermore, it is well established that adipocyte number and size remain largely constant after adolescence, with new adipocytes replacing old ones. Thus, differences in adipocyte number between lean individuals and those with obesity are likely established during childhood [20, 21]. These findings suggest that intrauterine life in term infants, or the period up to term‐equivalent age in preterm infants, may represent a critical window during which adipocyte number is established for life [22].

Given this background, we hypothesized that infants with LBW may exhibit early‐life adipose tissue maldevelopment, reflected in altered adipocyte size. Accordingly, the objective of this study was to evaluate adipocyte size during early childhood in infants with LBW, compare it with that in infants born appropriate for gestational age (AGA) at term (37–41 weeks' gestation), and determine the effects of adipocyte size on insulin sensitivity and adipocytokine levels, including adiponectin and leptin.

## 2 Methods

### 2.1 Subjects

The study protocol was approved by the Ethics Committee of Showa University School of Medicine (approval number: 2109), and written informed consent was obtained from the participants' parents. Participants were recruited from Showa University School of Medicine, Tokyo, Japan, between December 2015 and December 2019. Inclusion criteria were children aged 0.5–6.0 years who underwent surgery for an inguinal hernia, umbilical hernia, or testicular hydrocele. Children who underwent emergency or endoscopic surgery for inguinal hernia were excluded, as adipose tissue collection during these procedures could pose an additional risk. None of the participants had congenital anomalies or other major complications. Among the 97 participants recruited in this study according to the aforementioned inclusion criteria, two infants born large‐for‐date (defined as birth weight standard deviation [SD] score > 2 SD) and three preterm infants without LBW were excluded from the study population to allow comparison of adipocyte size between infants with LBW and infants born AGA at term (37–41 weeks' gestation), who served as non‐LBW controls. AGA was defined as a birth weight SD score between −2 SD and 2 SD, while SGA was defined as a birth weight SD score of ≤ −2 SD. Assuming 70 infants born AGA at term and 15 infants born LBW were included as study participants, and the mean adipocyte diameters (μm) during early childhood in infants born AGA at term and those with LBW were estimated at 75 ± 12 and 85 ± 12 μm, respectively, the statistical power was calculated to be 83.4%.

### 2.2 Methodology

Written informed consent was obtained from parents when the children were admitted to our hospital for surgery. General physical measurements, including body weight, length, and body mass index (BMI), were obtained preoperatively by trained nurses while participants wore light clothing without shoes. Waist circumference, triceps skinfold thickness, subscapular skinfold thickness, and arm circumference were carefully measured preoperatively by trained nurses with the participants wearing no clothing over the measurement sites. BMI was calculated as body weight divided by the square of height (kg/m^2^), and SD scores for body weight, body length, and BMI were determined according to the Japanese reference data.

Perinatal information, including gestational age, body weight, length, head circumference at birth, and body weight at 4 months of age, was also collected from the Japanese Mother–Child Health Handbook and birth records for all enrolled infants. Body weight SD score, length SD score, head circumference SD score at birth, and body weight SD score at 4 months of age were determined using Japanese reference data. Information on the duration of exclusive breastfeeding (≥ 6 months or not), the timing of introduction of complementary foods, and parental history of diabetes mellitus was obtained by administering a questionnaire and was confirmed via medical records.

Fasting blood samples were collected in the operating room before anaesthesia and centrifuged to obtain serum. Serum levels of glucose, insulin, triglycerides, total cholesterol, low‐density lipoprotein (LDL) cholesterol, high‐density lipoprotein (HDL) cholesterol, total adiponectin, leptin, and homeostatic model assessment of insulin resistance (HOMA‐IR) were measured. HOMA‐IR was calculated from fasting blood samples using the following formula: [HOMA‐IR = insulin (μU/L) × glucose (mg/dL)/405].

Subcutaneous adipose tissue samples (approximately 0.1 g) were obtained intraoperatively from the inguinal or abdominal regions of the participants. Intact pieces of each sample were immediately fixed for 48–72 h at 37°C in 25 mL of collidine buffer (pH 7.4) containing 2 g of osmium tetroxide/100 mL [23]. The osmium‐fixed specimens were washed with 0.01% Triton X‐100 and collected after incubation for 2–4 weeks at room temperature to allow adipocyte separation. The adipocyte diameter was measured directly under a microscope. For each sample, 100 adipocytes were randomly selected from multiple microscopic fields without consideration of cell size or morphology, to minimize sampling bias, and the mean diameter was calculated. Furthermore, the same observer repeated this procedure on three different occasions to calculate the intra‐observer coefficients for all 92 participants. The intra‐observer intra‐class coefficient for the mean adipocyte diameter was 0.978.

### 2.3 Statistical Analysis

Data analyses were performed using the statistical package for the social sciences (SPSS) Statistics Desktop for Japan Version 19.0 (IBM Company, Tokyo, Japan). Continuous variables are presented as the mean ± SD, except for insulin and HOMA‐IR, which are presented as median (interquartile range) because they were non‐normally distributed. Categorical data, such as sex and duration of exclusive breastfeeding (≥ 6 months or not), were analysed using Fisher's exact test. Continuous variables, including anthropometric measurements, blood parameters, and mean adipocyte diameter, were compared between infants with LBW and infants born AGA at term or between sexes using the Mann–Whitney U test, with the Hodges–Lehmann estimator used to estimate the 95% confidence interval (CI). Normality of adipocyte size distribution within each participant was assessed using the Shapiro–Wilk test. The correlation between mean adipocyte diameter and other variables was assessed using bivariate Pearson's correlation in a simple linear regression. Multiple regression analyses were conducted to evaluate differences in mean adipocyte diameter between infants with LBW and infants born AGA at term, adjusting for potential confounders, including sex, age, length SD score, BMI at surgery, duration of exclusive breastfeeding, and timing of complementary food introduction. Differences or associations were considered statistically significant at *p* < 0.05.

## 3 Results

The study included 92 children (77 born AGA at term and 15 with LBW), aged 0.8–5.2 years (76 males and 16 females). Their clinical characteristics at birth, 4 months of age, and during infancy are shown in Table 1. None of the parents had a history of diabetes mellitus. The LBW group (*n* = 15) included nine infants born preterm (60.0%; eight AGA and one SGA) and six infants born term (40.0%; three AGA and three SGA). Overall, four infants (26.7%) were classified as SGA. In the LBW group, the mean birth weight, birth weight SD score, and gestational age were 1928 g, −0.9 SD, and 34.9 weeks, respectively, compared with 3044 g, 0.2 SD, and 39.3 weeks in the term AGA group. Birth weight (95% CI: −1228, −835; *p* < 0.001), birth weight SD score (95% CI: −1.9, −0.6; *p* = 0.002), gestational age (95% CI: −4.15, −2.16; *p* < 0.001), length (95% CI: −6.3, −3.6; *p* < 0.001), length SD score (95% CI: −1.5, −0.4; *p* = 0.002), head circumference (95% CI: −4.0, −2.0; *p* < 0.001), and head circumference SD score (95% CI: −1.4, −0.4; *p* = 0.002) at birth were significantly lower in the LBW group than in the term AGA group. At 4 months of age, body weight (95% CI: −1.9, −0.8; *p* < 0.001) and body weight SD score (95% CI: −2.2, −0.8; *p* < 0.001) remained lower in the LBW group than in the term AGA group. The changes in body weight SD scores did not differ significantly between the two groups. The LBW group included fewer participants who were exclusively breastfed for ≥ 6 months than the AGA group (*p* = 0.045).

**TABLE 1:** Clinical characteristics of study participants at birth and during infancy.

	LBW group (*n* = 15)	Term AGA group (*n* = 77)	*p*	Total (*n* = 92)
*At birth*				
Sex (male/female)	12/3	64/13	n.s.	76/16
Gestational age (weeks)	34.9 ± 4.2	39.3 ± 1.2	< 0.001	38.6 ± 2.5
Body weight (g)	1928 ± 503	3044 ± 300	< 0.001	2862 ± 535
Body weight SD score	−1.1 ± 1.2	0.1 ± 0.7	0.002	−0.1 ± 0.9
Length (cm)	43.4 ± 5.0	49.6 ± 1.9	< 0.001	48.5 ± 3.5
Length SD score	−0.5 ± 0.9	0.6 ± 2.1	0.002	0.4 ± 2.0
Head circumference (cm)	30.0 ± 3.1	33.7 ± 1.4	< 0.001	33.1 ± 2.2
Head circumference SD score	−0.6 ± 0.9	0.4 ± 1.1	0.002	0.2 ± 1.1
*At the age of 4 months*				
Age (months)	4.2 ± 0.5	4.2 ± 0.4	n.s.	4.2 ± 0.4
Body weight (kg)	5.4 ± 1.0	6.8 ± 0.8	< 0.001	6.6 ± 1.0
Body weight SD score	−1.8 ± 1.6	−0.1 ± 0.8	< 0.001	−0.4 ± 1.2
Changes in body weight SD score^a^	−0.7 ± 2.0	−0.1 ± 0.8	n.s.	−0.1 ± 0.9
*During infancy*				
Exclusive breastfeeding ≥ 6 months (yes/no)	3/12	39/38	0.045	42/50
Start time of complimentary food (months)	6.5 ± 1.4	5.9 ± 0.5	n.s.	6.0 ± 0.8

*Note:* Categorial variables were described as numbers, and continuous variables were described as mean ± SD.

Abbreviations: AGA, appropriate gestational age; LBW, low birth weight; n.s., not significant; SD, standard deviation.

^a^ Between birth and the age of 4 months.

As shown in Table 2, the mean age at surgery was 2.5 years in the combined cohort, with no significant difference between the LBW and term AGA groups. The body weight SD score (95% CI: −1.6, −0.4; *p* = 0.001) and length SD score (95% CI: −1.8, −0.5; *p* = 0.001) at surgery were significantly lower in the LBW group than in the term AGA group; however, both groups had similar BMI and BMI SD scores. Furthermore, the waist circumference, triceps skinfold thickness, subscapular skinfold thickness, and arm circumference at the time of surgery did not differ between the two groups. The LBW group had significantly larger adipocyte size during early childhood compared with the term AGA group (mean adipocyte size: 87.0 μm vs. 74.7 μm; 95% CI: 5.2, 18.9; *p* = 0.002). A sensitivity analysis excluding the three infants born AGA at term from the LBW group confirmed the robustness of the findings. No significant difference in the mean adipocyte diameter was observed between the sexes.

**TABLE 2:** Anthropometric measurements, adipocyte size, and hematologic parameters of study participants at surgery.

	LBW group (*n* = 15)	Term AGA group (*n* = 77)	*p*	Total (*n* = 92)
Age (years)	2.3 ± 1.1	2.5 ± 1.1	n.s.	2.5 ± 1.1
Body weight (kg)	11.0 ± 2.7	12.7 ± 2.7	n.s.	12.4 ± 2.7
Body weight SD score	−0.9 ± 1.0	0.1 ± 0.9	0.001	0.0 ± 1.0
Length (cm)	83.1 ± 11.8	88.2 ± 9.7	n.s.	87.4 ± 10.2
Length SD score	−1.3 ± 1.4	−0.1 ± 0.9	0.001	−0.3 ± 1.1
Body mass index (kg/m^2^)	15.9 ± 1.3	16.2 ± 1.3	n.s.	16.1 ± 1.3
Body mass index SD score	0.0 ± 0.9	0.4 ± 1.0	n.s.	0.3 ± 1.0
Waist circumference (cm)	45.9 ± 3.8	46.9 ± 3.9	n.s.	46.7 ± 3.9
Triceps skinfold thickness (mm)	8.2 ± 3.5	8.4 ± 1.7	n.s.	8.4 ± 2.1
Subscapular skinfold thickness (mm)	5.7 ± 2.6	5.8 ± 1.7	n.s.	5.8 ± 1.8
Arm circumference (cm)	15.2 ± 1.2	15.7 ± 1.5	n.s.	15.7 ± 1.4
Mean adipocyte diameters (μm)	87.0 ± 12.9	74.7 ± 10.4	0.002	76.7 ± 11.7
Systolic blood pressure (mmHg)	96 ± 7	97 ± 7	n.s.	97 ± 7
Diastolic blood pressure (mmHg)	55 ± 6	56 ± 5	n.s.	56 ± 5
Insulin (μU/mL)	0.5 (0.3, 0.7)	0.7 (0.4, 1.1)	n.s.	0.6 (0.3, 1.1)
Glucose (mg/dL)	75 ± 17	83 ± 18	n.s.	81 ± 18
HOMA‐IR	0.10 (0.05, 0.12)	0.15 (0.07, 0.22)	n.s.	0.12 (0.06, 0.22)
Total adiponectin (μg/mL)	19.1 ± 4.7	18.9 ± 6.3	n.s.	18.9 ± 6.1
Leptin (ng/mL)	5.1 ± 1.8	5.2 ± 1.4	n.s.	5.2 ± 1.4
Total cholesterol (mg/dL)	156 ± 32	161 ± 28	n.s.	161 ± 28
LDL cholesterol (mg/dL)	94 ± 26	99 ± 24	n.s.	98 ± 25
HDL cholesterol (mg/dL)	53 ± 7	54 ± 13	n.s.	54 ± 12
Triglyceride (mg/dL)	49 ± 24	51 ± 28	n.s.	51 ± 28

*Note:* Insulin and HOMA‐IR are presented as medians with interquartile range, whereas other continuous variables were presented as means ± SD.

Abbreviations: AGA, appropriate‐for‐gestational‐age; HDL, high‐density lipoprotein; HOMA‐IR, homeostatic model assessment of insulin resistance; LBW, low birth weight; LDL, low‐density lipoprotein; n.s., not significant; SD, standard deviation.

**FIGURE 1:**
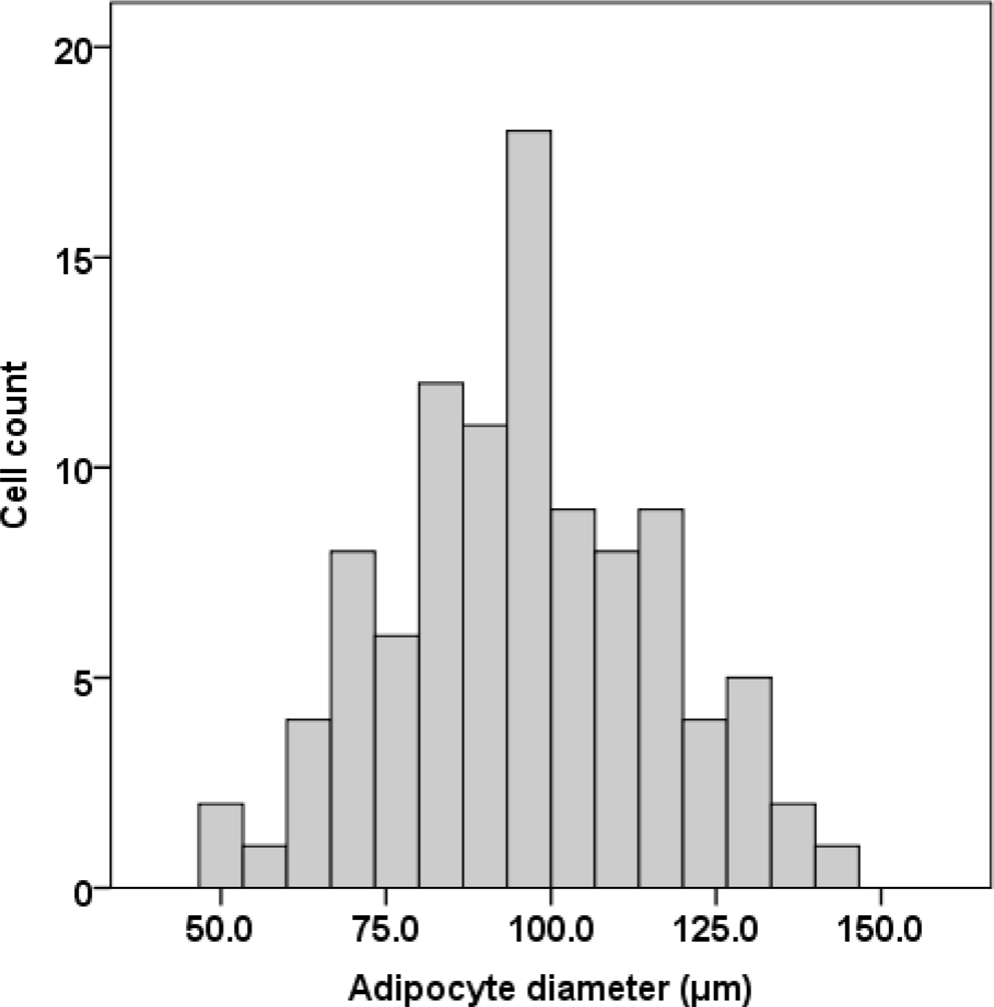
Frequency distribution of adipocyte diameters. Adipocyte diameters were approximately normally distributed, without evidence of skewness or bimodality.

Figure 1 shows the frequency distribution of adipocyte sizes from a representative infant in the LBW group (born at 35.3 weeks of gestation with a birth weight of 1625 g). The adipocyte sizes appear to be normally distributed, without evidence of skewness or bimodality. Furthermore, the null hypothesis of normality was not rejected in the majority of cases in both groups based on the Shapiro–Wilk test.

The LBW group had lower insulin levels (95% CI: −0.44, 0.03; *p* = 0.090) and HOMA‐IR (95% CI: −0.100, 0.013; *p* = 0.090) than the term AGA group, although these differences were not statistically significant. None of the other haematological parameters, including glucose, triglycerides, total cholesterol, LDL cholesterol, HDL cholesterol, total adiponectin, and leptin, differed between the LBW and term AGA groups. Simple regression analyses in the combined cohort revealed that the mean adipocyte diameter was not associated with circulating metabolic markers, including insulin, HOMA‐IR, adiponectin, and leptin.

In the combined cohort, simple regression analyses showed that mean adipocyte diameter was positively associated with BMI (*r* = 0.494, *p* < 0.001), BMI SD score (*r* = 0.461, *p* < 0.001), subscapular skinfold thickness (*r* = 0.208, *p* = 0.047), and triceps skinfold thickness (*r* = 0.334, *p* = 0.001) at surgery. In contrast, it was negatively associated with gestational age (*r* = −0.224, *p* = 0.032), birth weight (*r* = −0.326, *p* = 0.002), birth weight SD score (*r* = −0.224, *p* = 0.032), length (*r* = −0.363, *p* < 0.001) and length SD score (*r* = −0.240, *p* = 0.021) at birth, and length SD score at surgery (*r* = −0.306, *p* = 0.003). The mean adipocyte diameter was not significantly associated with head circumference and head circumference SD score at birth, waist circumference, or arm circumference at surgery.

As shown in Table 3, multiple regression analysis revealed that LBW remained a significant determinant of the mean adipocyte diameter after adjusting for variables such as sex, age, length SD score, BMI at surgery, duration of exclusive breastfeeding, and timing of complementary food introduction (*p* < 0.001). Similarly, BMI at surgery remained a significant determinant of the mean adipocyte diameter in the multiple regression analysis (*p* < 0.001).

**TABLE 3:** Multiple regression analysis of factors related to mean adipocyte diameters during early childhood.

	Mean adipocyte diameter	
	Standardized β	*p*
Sex (male)	0.098	0.245
Age	0.158	0.072
Low birth weight	0.433	< 0.001
Body mass index at the age of surgical operation	0.557	< 0.001
Length SD score at the age of surgical operation	−0.123	0.186
Exclusive breastfeeding for ≥ 6 months	0.137	0.108
Start time of complimentary food	0.016	0.848

*Note:* Adjusted *R* ^2^ = 0.441, *p* < 0.001.

Abbreviation: SD, standard deviation.

## 4 Discussion

The present study demonstrated that infants with LBW had larger adipocytes during early childhood than infants born AGA at term, despite lower body weight and length SD scores and similar BMI. Multiple regression analysis revealed that both LBW and a higher BMI at the time of surgery were significant predictors of larger adipocyte size during early childhood. To the best of our knowledge, this study is the first to demonstrate early adipocyte enlargement in infants with LBW, even in the absence of obesity, highlighting the potential for an increased risk of insulin resistance development in later life.

The distribution pattern of adipocyte size observed in this study suggests that adipocyte enlargement in infants with LBW reflects a generalized increase in cell size across the adipocyte population, rather than the presence of a distinct subpopulation of enlarged adipocytes. This contrasts with patterns reported in obesity, where adipocyte size distributions are often skewed or bimodal, indicating heterogeneity in adipocyte populations [24, 25]. In our exploratory analysis of the combined cohort, adipocyte size was not significantly associated with circulating metabolic markers, which supports this interpretation. These findings suggest the notion that adipocyte enlargement in early childhood may represent a structural or developmental characteristic rather than a pathological state.

Nonetheless, the factors underlying larger adipocytes in infants with LBW during early childhood remain unclear. One possible explanation is that infants with LBW may exhibit adaptive metabolic responses, including features consistent with the ‘thrifty phenotype’, such as altered endocrine and metabolic regulation associated with impaired growth potential and reduced muscle mass [21]. In this context, increased lipid storage in adipocytes during early postnatal life has been described as ‘catch‐up fat’ [26, 27]. However, these mechanisms were not directly assessed in the present study and therefore should be interpreted with caution.

Furthermore, adipocyte number is thought to be largely established during fetal and early postnatal development through the balance between precursor cell proliferation, differentiation, and adipocyte hypertrophy [21]. Within this framework, alterations in adipocyte precursor cell pools may influence adipose tissue expandability. Infants with LBW, who are frequently exposed to undernutrition during intrauterine life (in term infants) and/or early postnatal life (in preterm infants), may exhibit impaired adipocyte hyperplasia, reflected in a reduced number of adipocytes. This may lead to increased lipid load per cell and compensatory adipocyte hypertrophy. This concept is consistent with our previously proposed ‘overloaded adipocyte hypothesis’ [22]. This may partly explain why infants with LBW exhibit larger adipocytes than infants born AGA at term, despite having similar BMI in the present study.

Notably, infants with LBW had lower serum insulin levels and HOMA‐IR values than infants born AGA at term, although the differences were not statistically significant. This may reflect the developmental stage of early childhood, which is characterized by rapid growth, high energy demand, and generally increased insulin sensitivity. In this context, larger adipocytes may not necessarily indicate metabolic dysfunction. Given that adipocyte size reflects triglyceride accumulation, it is influenced by the balance between lipid storage and mobilization, processes regulated in part by insulin signalling [28, 29, 30]. Considering the relatively modest mean adipocyte diameter in the LBW group, adipocyte hypertrophy at this stage may represent a developmental or structural phenotype rather than overt metabolic impairment, consistent with a previous study suggesting that early characteristics of adipose tissue may be associated with later metabolic risk [31]. Taken together, these findings suggest that early adipocyte enlargement in infants with LBW may reflect altered adipose tissue development and limited expandability, potentially representing an early structural phenotype associated with later metabolic risk. However, further longitudinal and functional studies are required to clarify these mechanisms.

The limitations of this study need to be addressed. First, the study lacked sufficient statistical power to evaluate sex differences, owing to the limited number of female participants. In the subcutaneous white adipose tissue, adipocytes were shown to preferentially undergo hypertrophy in response to a high‐fat diet in male mice, whereas female mice exhibited adipocyte hyperplasia [32]. Second, the LBW group in the current study included both growth‐restricted and preterm infants, which may have influenced our results. We previously investigated longitudinal changes in adiponectin multimers in term and preterm infants between birth and 12 months of age [33, 34, 35, 36]. These findings suggest that fat cell hypertrophy may occur to some extent between birth and term‐equivalent age in preterm infants, but not in term infants. Prematurity and fetal growth restriction may differentially influence adipose tissue development through distinct biological mechanisms. Therefore, whether adipose tissue maldevelopment, including altered adipocyte size, is comparable between infants with LBW due to growth restriction and those with LBW due to prematurity remains unclear. However, further stratified analyses were not feasible due to the limited sample size of the LBW group. Moreover, the strong collinearity between birth weight and gestational age makes it difficult to disentangle their independent effects. These issues should be addressed in future studies with larger sample sizes. Third, subcutaneous adipose tissue samples were collected from different anatomical depots (inguinal or abdominal regions), depending on the surgical indication. As adipocyte size may vary by depot, thus, the potential influence of depot‐specific variation cannot be excluded.

In conclusion, infants with LBW had larger adipocytes than infants born AGA at term during early childhood, even after adjusting for confounding factors, such as BMI. Further studies are warranted to determine whether early adipocyte hypertrophy contributes to the development of insulin resistance later in life.

## Funding

This work was supported by the Japan Society for the Promotion of Science (JP16K19695).

## Conflicts of Interest

The authors declare no conflicts of interest.

## Data Availability

The data that support the findings of this study are available on request from the corresponding author. The data are not publicly available due to privacy or ethical restrictions.
